# Accelerometry-Based Assessment of Overnight Coat Use on Dog Sleep and Activity Patterns: Implications for Farm Dog Welfare

**DOI:** 10.3390/ani16132035

**Published:** 2026-07-02

**Authors:** Ting Wang, Michelle Smit, Xuan Cai, Rene A. Corner-Thomas, Ina Draganova, Christopher J. Andrews, David G. Thomas

**Affiliations:** 1 School of Agriculture and Environment, Massey University, Palmerston North 4442, New Zealand; wendy710119@gmail.com (T.W.); m.smit@massey.ac.nz (M.S.); i.draganova@massey.ac.nz (I.D.); c.j.andrews@massey.ac.nz (C.J.A.); d.g.thomas@massey.ac.nz (D.G.T.); 2 Institute of Animal Husbandry and Veterinary Science, Shanghai Academy of Agricultural Sciences, Shanghai 201403, China; caixuan1985911@163.com

**Keywords:** canine, working farm dogs, thermoregulation, sleep behaviour, accelerometry, nutrient digestibility

## Abstract

New Zealand working farm dogs can struggle to maintain their body weight and condition during periods of high workload, despite substantial energy intake, highlighting the need to optimise energy balance. One potential strategy for reducing overall energy demands is to reduce energy expenditure for thermoregulation, particularly during colder conditions, which may improve both recovery and energy utilisation. This study investigated whether wearing coats influenced the sleep behaviour, activity, and apparent nutrient digestibility of outdoor-kennelled dogs. Eight adult working-breed dogs (*n* = 8) were studied using a randomised cross-over design, in which dogs wore a coat or no coat during overnight periods (15:00–09:00 h). Behaviour was monitored using triaxial accelerometers and classified using a validated machine learning model, while apparent nutrient digestibility was determined from pooled faecal samples. Dogs spent more time sleeping when wearing a coat compared to no coat (48.1% vs. 40.0%, *p* = 0.008), with the effect being most evident during cooler evening hours. Time spent resting and being active was reduced when coats were worn (*p* < 0.05), while overall activity did not differ (*p* = 0.856). No differences were observed in apparent digestibility of energy or nutrients (*p* > 0.05), although protein digestibility tended to be higher when coats were worn (*p* = 0.079). These findings suggest that coats can improve sleep behaviour, likely through improved thermal comfort. While this study was conducted in research dogs under relatively mild temperature conditions, the results indicate the potential welfare and functional benefits for working farm dogs.

## Simple Summary

This study looked at whether putting coats on dogs overnight could help improve their energy use and wellbeing, especially in cooler conditions. Eight adult dogs were tested using a cross-over design where they alternated between wearing a coat and not wearing one. When the dogs wore coats, they spent more time sleeping (48% vs. 40%) and less time resting or being active, particularly during cooler evening hours. However, their overall activity levels stayed about the same. This suggests that coats helped the dogs feel more comfortable and settle more easily. There were no clear differences in how well the dogs digested energy or nutrients. While the study was conducted under mild conditions, the findings suggest that coats could benefit working farm dogs by supporting rest and potentially improving their recovery and energy balance during demanding periods.

## 1. Introduction

Working farm dogs, primarily Huntaways and Heading dogs, are a vital component of New Zealand’s agricultural industry [1]. It has been estimated that there are approximately 200,000 farm dogs in New Zealand [2]. Many farms in New Zealand, especially sheep and beef units, rely heavily on farm dogs to help move and manage stock [3]. According to Arnott et al. [4], the average shepherd dog can contribute $40,000 in value over its lifetime, while costing only $7700 to feed and maintain.

New Zealand farm dogs are characterised by their high workload, travelling average distances of at least 20 km per day and working for approximately 9 h during peak periods [3]. The intensity of work of farm working dogs is comparable to that of sled dogs, with a mean daily energy requirement of up to 202 kcal/kg BW^0.75^ during peak periods [3]. In contrast, pet dogs have a mean daily energy requirement of 123.8 kcal/kg BW^0.75^ [5]. To maintain body weight and support high workloads, working dogs require a higher energy intake than pet dogs. Despite this, many working dogs in New Zealand have body condition scores below what is considered “ideal” for pets, and farmers report being unable to provide sufficient energy to maintain optimal condition during extended periods of work [6].

There is limited research on how environmental conditions, specifically ambient temperature, affects the overall health of New Zealand farm dogs, although some evidence suggests that ambient temperature can influence health and physiological outcomes [7]. For dogs, the range of ambient temperatures in which minimal energy is required for thermoregulation (the thermoneutral zone) is between 20 and 30 °C [8]. Outside this range, energy requirements increase to maintain core body temperature [9,10]. Insulative properties, such as coat length and density, play an important role in determining how effectively dogs can maintain their thermal balance, with longer-haired dogs showing smaller increases in energy requirements under cold conditions compared to short-haired dogs [11,12].

Despite substantial energy intake, some farm dogs struggle to maintain their body weight during peak work periods [6]. Therefore, optimising energy balance is important, either by reducing energy expenditure or improving dietary energy utilisation. One potential strategy is to minimise the energy required for thermoregulation, particularly during winter. While the energy cost of work cannot be readily reduced, providing external insulation through wearable coats may lower heat loss and reduce overall energy demands. Cold conditions can increase thermoregulatory requirements, which may disturb rest and elevate total energy expenditure [9,13]. As rest and sleep are associated with energy conservation and metabolic regulation, improving thermal comfort may support more consistent sleep patterns and help limit unnecessary energy loss. This may also support metabolic efficiency by allowing greater allocation of energy to maintenance and recovery processes such as tissue repair [14,15]. This study therefore aimed to determine whether wearing a coat overnight could improve the sleep duration and apparent nutrient digestibility of New Zealand working dogs kennelled outside.

## 2. Materials and Methods

The manipulations in this study were approved by the Massey University Animal Ethics Committee (Protocol 24/38) under the New Zealand Animal Welfare Act (1999) [16]. The study was carried out between 7–17 October 2024 at the Massey University Canine Nutrition Unit located in Palmerston North, New Zealand (latitude 40°23′30″ S longitude 175°36′58″ E).

### 2.1. Animals and Animal Husbandry

Eight desexed dogs of farm working dog breeds (four males and four females), aged 5.75 ± 0.81 years, and weighing 25.01 ± 1.01 kg, were used for this study (Table 1). All dogs were single-coated and short-haired. Dogs were fed their standard complete and balanced diet (Black Hawk Working Dog, Masterpet Corporation Ltd., Lower Hutt, New Zealand; Table 2). Daily feed allowances were based on weekly body weight measurements and calculated using the following equation: ME Requirement = 125 × Body Weight (kg)^0.75^ [9]. Dogs received their daily food allowance at 1600 h each day and had ad libitum access to water, which was refreshed daily.

The dogs were housed individually in one of eight semi-sheltered pens (measuring 4 m × 3 m). Dogs remained in the same pen throughout the study period, with the neighbouring animals kept consistent. The building had open sides, a solid concrete floor and was covered with a metal sheet roof, allowing exposure to natural light and environmental conditions. Each run was equipped with a kennel (70 cm × 65 cm × 70 cm) with a blanket for bedding. Although microclimatic conditions were not recorded at the individual pen level, all pens were located within the same structure and were therefore assumed to be exposed to similar environmental conditions. The pens were washed out daily using a high-pressure hose. All dogs were walked on a lead daily for 30 min.

### 2.2. Experimental Design

**Figure 1:**
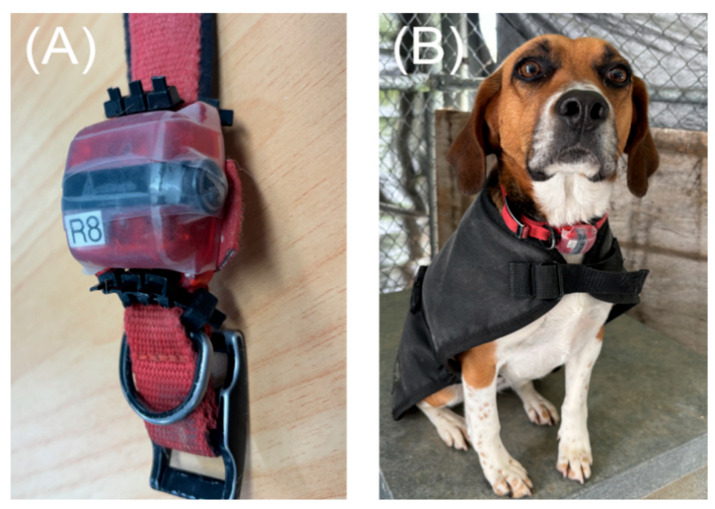
Accelerometer placement and coat fitting showing (**A**) a close-up view of the accelerometer wrapped in waterproof tape and fixed to a collar, and (**B**) a dog fitted with the coat and collar-mounted accelerometer.

Prior to commencing the study, each dog was fitted with a collar-mounted ActiGraph^®^ accelerometer (WGT3X-BT, ActiGraph, Pensacola, FL, USA, weighing 19 g and measuring 33 mm × 46 mm × 15 mm; Figure 1A). These devices measured raw acceleration data along three independent axes (lateral, craniocaudal, and dorsoventral) at 30 Hz with a dynamic range of ±8.0 m/s^2^. Accelerometry was selected as a minimally invasive approach that enabled continuous, objective quantification of activity–rest patterns in the free-living animals without disrupting normal behaviour, and was less labour-intensive than manual scoring of behaviour. Acceleration data were collected continuously throughout the ten-day study period, after which the data were downloaded, and the behaviours of the dogs were classified using a validated machine learning algorithm [18] (see Section 2.3).

The study used a randomised cross-over block design balanced for body weight and sex, with dogs allocated to one of two treatment sequences (Table 1). The study consisted of two experimental phases. In phase 1 (study days 0–5), dogs assigned to the coat-first sequence wore a coat (Natural Hound, Ongaonga, Hawke’s Bay, New Zealand; Figure 1B) from 15:00 h to 09:00 h for five consecutive days, while dogs assigned to the no-coat-first sequence did not wear a coat. In phase 2 (study days 5–10), the treatments were reversed. In each phase, a one-day acclimatisation period preceded four days of data collection. Periods were classified according to a coat-associated time window, defined as the coat window (15:00–09:00 h) and the non-coat window (09:00–15:00 h).

Faeces were collected daily from each dog at 10:00 h and 15:30 h each day throughout the experiment. Samples were pooled by dog within each experimental phase and stored at −20 °C until analysis for apparent nutrient digestibility.

Hourly weather data, including temperature (°C), wind speed (m/s), and rainfall (mm), were downloaded from the National Climate Database of the National Institute of Water and Atmospheric Research [19] using data collected by the Palmerston North EWS weather station, agent number 21963 (latitude 40°22′55.02″ S, longitude 175°36′32.94″ E) located within 2 km of the study site.

### 2.3. Accelerometry and Behaviour

At the end of the study, the raw acceleration data were downloaded from the devices using the proprietary software ActiLife 6^®^ (version 6.13.4; ActiGraph, Pensacola, FL, USA) and exported as comma-separated value (CSV) files. The acceleration data were then processed into behavioural data using a validated random forest model [18]. The selected model classified behaviour into one of eight categories and had an overall accuracy of 74%, balanced accuracy of 83%, an F1-score of 78%, and a kappa coefficient of 0.68. The balanced accuracies of each behaviour were as follows: barking (90%), drinking (86%), eating (86%), locomotion (77%), resting-asleep (sleeping; 94%), resting-alert (resting; 89%), sniffing (95%), and standing (72%; [18]). For the purpose of this study, drinking and eating behaviour were excluded, and barking, locomotion, sniffing and standing were merged into “active”. Resting-asleep, henceforth referred to as “sleeping,” was defined as a recumbent posture with the head lowered and supported on the ground, limbs, or a surface, including both sternal lying (head down) and lateral recumbency. Resting-alert, henceforth referred to as “resting,” was defined as a stationary posture with the body supported either in a sitting position or sternal lying with the head held up (i.e., not supported by the ground, a surface or limbs). See [19] for more detailed definitions of all behaviours as classified by the model.

Overall dynamic body acceleration (ODBA) was also extracted independently from the raw acceleration data and analysed separately as a measure of overall activity. Overall dynamic body acceleration reflects the intensity and magnitude of body movements over time, providing an indication of how active an animal is. The ODBA was calculated as the sum of the absolute values of dynamic body acceleration (DBA) across the three axes (Equation (1)). The DBA was derived by subtracting a 1 s moving average (30 datapoints) from the raw acceleration signal for each axis.

(1) $$ODBA=\sum_{t=1}^{N}\left|{DBA}_{x}\right|+\left|{DBA}_{y}\right|+\left|{DBA}_{z}\right|$$

### 2.4. Apparent Nutrient Digestibility

After completion of the study, faecal samples were freeze-dried (FD600GPC, Cuddon Engineering Ltd., Blenheim, New Zealand), the total weight of each sample was recorded, and then the samples were ground. For each phase of the experiment, the faecal samples of each dog were pooled. A subsample (50 g) of the pooled faecal sample was then taken and analysed in duplicate, along with a ground sample of the diet, at the Massey University Nutrition Laboratory (College of Sciences, Palmerston North, New Zealand).

The diet and faecal samples were analysed to assess the amount of dry matter (DM; AOAC 930.15) [20], crude ash (AOAC 942.05) [21], crude protein (AOAC 992.15) [22], and crude fibre (AOAC 978.19) [23]. Crude fat was determined according to the Mojonnier (AOAC 922.06) [24] and Soxtec (AOAC 2003.06) [25] methods for the diet and faecal samples, respectively. Gross energy was determined by bomb calorimetry using a Gallenkamp Autobomb (London, UK) according to the procedure recommended by the manufacturer. The apparent digestibility of dry matter, crude fat, crude protein, and gross energy was calculated according to Equation (2):

(2) $$Apparent\;Digestibility\;(\%)=\left(\frac{Nutrient\;Intake-Nutrient\;in\;Faeces}{Nutrient\;Intake}\right)\times 100$$

### 2.5. Statistical Analyses

All data processing and statistical analyses were carried out using R statistical software, version 4.1.1 [26]. Prior to statistical analyses, all data were assessed for normality using Shapiro–Wilk tests and visual inspection of Q-Q plots. The results were considered significant at *p* < 0.05, with 0.05 ≤ *p* < 0.10 considered as a trend.

Behavioural time budgets were expressed as the proportion of time spent performing each behaviour and analysed using generalised linear mixed models (GLMMs) fitted with the “glmmTMB” package (version 1.1.12) [27]. Analyses included time spent active, resting, and asleep, along with ODBA as a continuous measure of activity.

To assess treatment (coat vs. no coat) effects, mean daily behavioural proportions were calculated for each animal (*n* = 8) under each treatment (coat vs. no coat). Separate beta-distributed GLMMs with a logit link were fitted for each behaviour, with treatment included as a fixed effect and animal included as a random effect to account for repeated measures. Mean daily ODBA was analysed using a gamma-distributed GLMM with a log link. Additional models were then used to assess whether daily behaviour and hourly ODBA varied according to time relative to the coat-wearing period. These models included treatment, coat exposure window, and their interaction as fixed effects, with animal included as a random effect.

Model simplification was performed using backward stepwise removal of fixed effects where *p* > 0.10. Marginal and conditional R^2^ values were calculated for final models and, where appropriate, post hoc pairwise comparisons were performed using estimated marginal means with false discovery rate (FDR) correction. For all GLMMs, fixed-effect estimates are reported as β ± SE on the link scale and treatment effects are visualised using estimated marginal means and 95% confidence intervals.

Finally, apparent digestibility coefficients (gross energy, dry matter, crude protein, crude fat) were analysed using paired *t*-tests and are presented as mean ± SE.

## 3. Results

**Figure 2:**
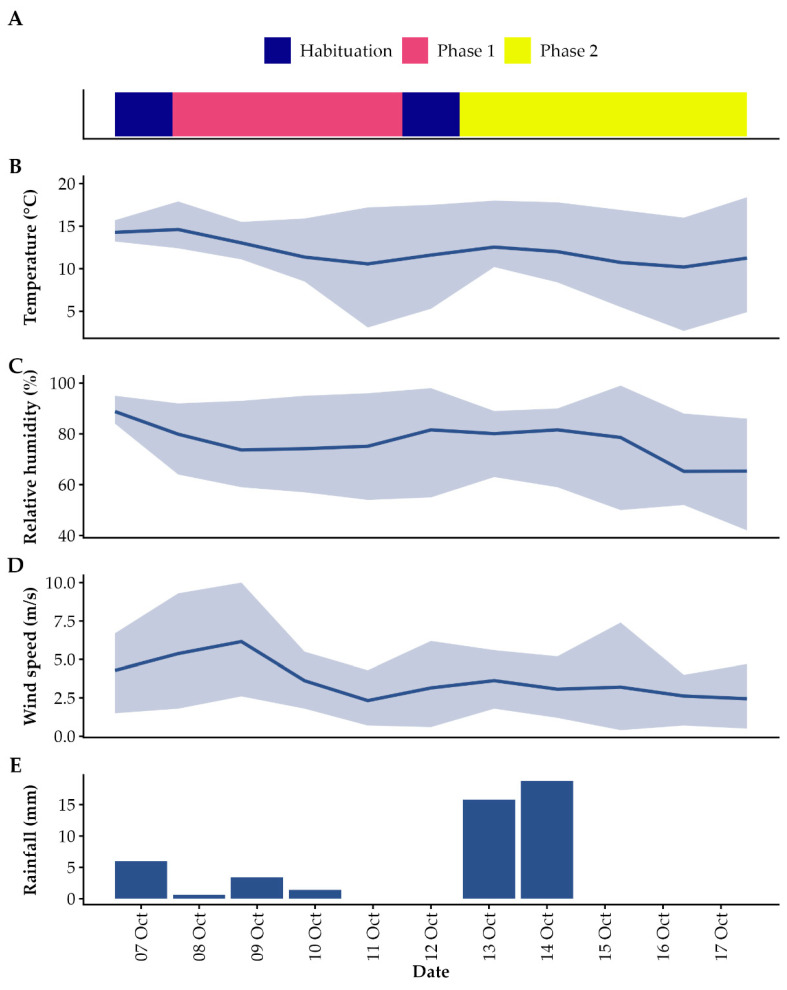
Daily environmental conditions during the study period. Panels show (**A**) phases of the study, (**B**) mean daily ambient temperature (°C), (**C**) mean daily relative humidity (%), (**D**) mean daily wind speed (m/s), with shaded areas indicating daily minimum and maximum values, and (**E**) total daily rainfall (mm).

During the study period (7 October to 17 October 2024), the ambient temperature ranged from 2.7 to 18.4 °C with a daily mean of 12.0 °C (Figure 2). The mean relative humidity was 76.7%, with values ranging from 42.0% to 99.0%. The total rainfall over the study period was 46 mm and the mean wind speed was 3.6 m/s, with recorded values ranging from 0.4 to 10.0 m/s.

### 3.1. Behaviour and ODBA

No adverse responses to wearing the coats were observed in the dogs. On average, dogs were observed to sleep for 11 h and 6 min (±47 min) per day, while they rested for 5 h and 40 min (±26 min). Dogs were active for 7 h and 12 min ± 24 min.

#### 3.1.1. Coat vs. No Coat

**Figure 3:**
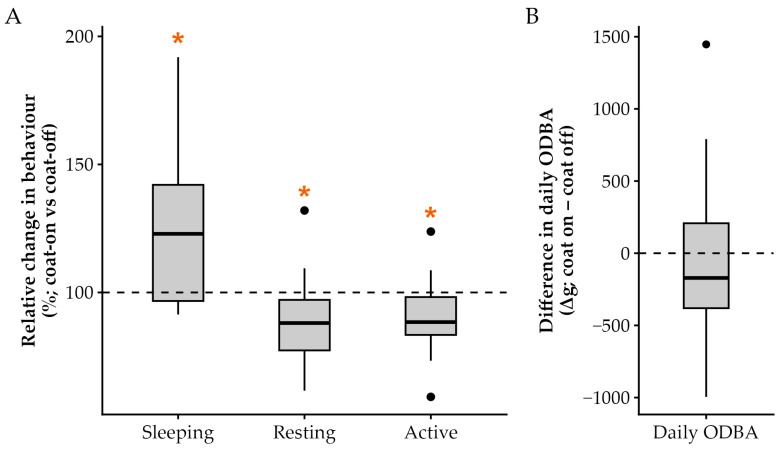
(**A**) Relative change in observed time spent on sleeping, resting and active behaviours when wearing a coat compared to no coat (coat-on relative to coat-off; %), calculated per dog. (**B**) Difference in observed daily ODBA between treatments (coat–no coat; Δg), calculated per dog. The dashed line at 100% indicates no change. Black circles indicate outlier observations, defined as values more than 1.5 times the interquartile range beyond the boxplot whiskers. Values above 100% indicate an increase with coat, and values below 100% indicate a decrease. Asterisks indicate a significant treatment effect resulting from the GLMM.

Treatment was significantly associated with differences in sleeping, resting and active behaviours. The proportion of time spent sleeping differed between treatments (χ^2^ = 7.11; *p* = 0.008), with a positive coat-on effect (β = 0.33 ± 0.15 SE). The proportion of time spent sleeping was higher when dogs wore a coat (48.1%, 95% CI: 41.6–54.7) compared to when they did not (40.0%, 95% CI: 33.8–46.4; Figure 3). The fixed effect of treatment explained 25.0% of the variance in sleeping behaviour, whereas the full model, including treatment and the random effect of dog, explained 83.4%.

Similarly, the amount of time spent resting differed between treatments (χ^2^ = 6.34, *p* = 0.012), with a negative coat-on effect (β = −0.19 ± 0.09 SE). The proportion of time spent resting was lower when the dogs were wearing a coat (22.5%, 95% CI: 17.9–27.8) compared to when they did not wear a coat (25.9%, 95% CI: 20.9–31.6; Figure 3A). The treatment effect explained 5.6% of the variance in resting behaviour and the full model explained 92.7%.

The proportion of time spent showing active behaviours also differed between treatments (χ^2^ = 4.98, *p* = 0.026), with a negative coat-on effect (β = −0.21 ± 0.10 SE). The proportion of time spent showing active behaviours was lower when the dogs were wearing the coats (28.2%, 95% CI: 22.9–34.3) compared to when they did not wear a coat (32.7%, 95% CI: 26.9–39.1; Figure 3A). The treatment effect explained 26.1% of the variance in active behaviours and the full model explained 76.1%.

For daily ODBA, a small negative coat-on effect was observed (β = −0.01 ± 0.07 SE), but there was no significant difference between treatments (χ^2^ = 0.03, *p* = 0.856). Daily ODBA values were similar when dogs wore the coats (5464 Δg, 95% CI: 4174–7154) compared to when they did not (5531 Δg, 95% CI: 4225–7240; Figure 3B). The treatment effect explained 0% of the variance and the full model explained 88.3%.

An overview of both the observed and GLMM predicted means of time spent showing different behaviours and the daily ODBA can be found in Appendix A.

#### 3.1.2. Coat vs. No Coat and Coat-Associated Time Window

Sleeping behaviour showed a trend for an interaction between treatment and the coat-associated time window (χ^2^ = 2.84, *p* = 0.092) and was therefore retained in the final model. During the non-coat window, dogs spent little time sleeping, and this did not differ between treatments (coat-on effect: β = −0.12 ± 0.28 SE, *p* = 0.676). Dogs spent 8.8% (95% CI: 5.7–13.2) of their time sleeping when wearing their coats during the coat window compared to 9.8% (95% CI: 6.5–14.4) when not wearing a coat during the coat window. However, the amount of time spent sleeping increased during the coat window (β = 2.20 ± 0.23 SE, *p* < 0.001), averaging at 49.4% (95% CI: 41.2–57.6) when not wearing a coat and 60.1% (95% CI: 51.8–67.8) when wearing a coat. Within each treatment, dogs spent a greater predicted proportion of time sleeping during the coat window than during the non-coat window (*p* < 0.001). During the coat window, dogs spent a greater proportion of time sleeping when wearing a coat compared to the dogs that were not wearing a coat (*p* = 0.016), consistent with a positive treatment resulting from the coat window interaction effect (β = 0.55 ± 0.33 SE). In the final model, the fixed effects of treatment, coat window, and their interaction explained 90.4% of the variance in sleeping behaviour, whereas the inclusion of the random effect “dog” increased the explained variance to 96.5%. An overview containing both the observed and GLMM predicted means of time spent showing sleeping behaviours can be found in Appendix B.

Resting behaviour showed no significant interaction between treatment and coat-associated time window (*p* = 0.546) and the interaction term was therefore removed from the model. Resting behaviour was significantly associated with the coat-associated time window, which was retained as the sole fixed effect in the final model (χ^2^ = 64.57, *p* < 0.001). Resting time decreased during the coat window, as indicated by a negative effect on the logit scale (β = −1.45 ± 0.18 SE). Dogs spent a substantially greater proportion of time resting during the non-coat window (47.0%, 95% CI: 37.4–56.9) compared with the coat window (17.2%, 95% CI: 11.7–24.5). In the final model, the fixed effect of coat-associated time window explained 66.5% of the variance in resting behaviour, whereas the inclusion of the random effect of dog increased the explained variance to 95.0%. An overview containing both the observed and GLMM predicted means of time spent showing resting behaviour can be found in Appendix C.

Active behaviours showed no significant interaction between treatment and coat-associated time window (*p* = 0.224), and the interaction term was therefore removed from the model. Active behaviour was significantly associated with the coat-associated time window, which was retained as the only fixed effect in the final model (χ^2^ = 6.29, *p* = 0.012). Active behaviours decreased during the coat window, as indicated by a negative effect on the logit scale (β = −0.68 ± 0.27 SE). Dogs spent a greater proportion of time active during the non-coat window (43.1%, 95% CI: 34.7–52.0) compared with the coat window (27.8%, 95% CI: 20.7–36.3). The fixed effect explained 52.0% of the variation in active behaviour. An overview containing both the observed and GLMM predicted means of time spent showing active behaviours can be found in Appendix D.

Hourly ODBA showed a trend towards an interaction between treatment and coat-associated time window (χ^2^ = 3.34, *p* = 0.068) and was therefore retained in the final model. During the non-coat window, hourly ODBA did not differ between treatments (coat-on effect: β = 0.08 ± 0.07 SE, *p* = 0.266), with similar estimated values when dogs were not wearing a coat (503 Δg, 95% CI: 383–663) compared to when they were wearing a coat (546 Δg, 95% CI: 415–718 Δg). In contrast, hourly ODBA was markedly lower during the coat-associated window (β = −1.09 ± 0.07 SE on the log scale, *p* < 0.001), averaging 170 Δg (95% CI: 129–223) when dogs were not wearing a coat and 153 Δg (95% CI: 116–201) when dogs were wearing a coat. Within each treatment, dogs exhibited significantly lower hourly ODBA during the coat than the non-coat window (pairwise comparisons, both *p* < 0.001). During the coat window, no significant difference in hourly ODBA was detected between dogs wearing a coat and those not wearing a coat (*p* = 0.169), consistent with the negative but non-significant treatment × window interaction effect (β = −0.19 ± 0.10 SE). In the final model, the fixed effects of treatment, coat window, and their interaction explained 69.8% of the variance in resting behaviour, whereas the inclusion of the random effect of dog increased the explained variance to 96.0%. An overview containing both the observed and GLMM predicted means’ hourly ODBA can be found in Appendix E.

#### 3.1.3. Daily Pattern in Sleeping Behaviour and ODBA

**Figure 4:**
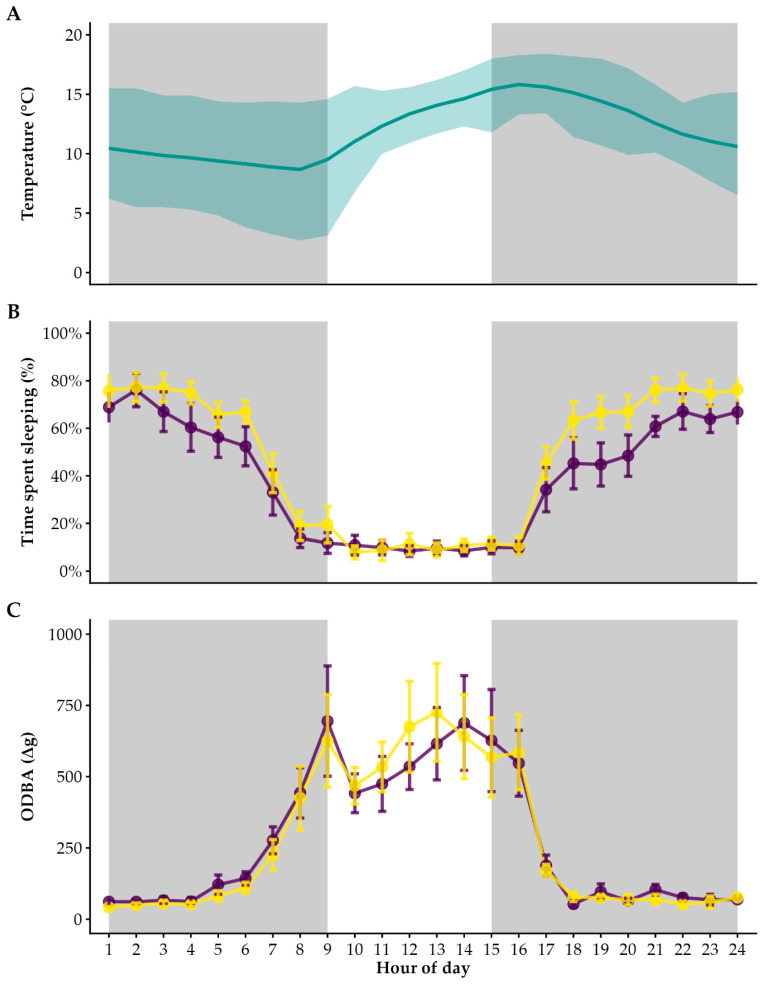
Daily patterns of (**A**) ambient temperature (°C; mean with shaded area indicating the average minimum and maximum), (**B**) time spent sleeping (%), and (**C**) dynamic body acceleration (ODBA; Δg), comparing periods when dogs wore coats (yellow) and when no coat was worn (purple). The coat-associated window is indicated across all panels in grey. In panels (**B**,**C**), points represent hourly means and error bars indicate the standard error (±SE).

Sleep behaviour and activity followed a diurnal pattern aligned with both environmental conditions and the daily routine within the facility (Figure 4). Dogs spent the most time sleeping (50–80%) between 17:00 h and 07:00 h, coinciding with both sunset around 17:30 h and staff leaving. Sleep declined sharply from 07:00 h, around 30 to 60 min prior to staff arriving, with minimal levels (~10%) of sleep between 09:00 h and 16:00 h. When dogs wore a coat during the coat window, they generally spent more time sleeping compared to when they did not wear a coat, with this difference being more pronounced between 19:00 h and 21:00 h. Activity, measured as ODBA, showed an inverse pattern to sleeping, whereby the lowest levels were observed between 18:00 h and 06:00 h (~50–125 Δg). Activity increased quickly from 07:00 h (~250 Δg) until 09:00 h (~660 Δg) and was highest between 09:00 h and 16:00 h (~455–660 Δg). Activity sharply declined between 16:00 h (~566 Δg) and 18:00 h (~65 Δg) and was lowest from 18:00 h till 06:00 h (~50–130 Δg). This pattern coincided with the daily temperature cycle shown in Figure 4A.

### 3.2. Digestiblity

**Figure 5:**
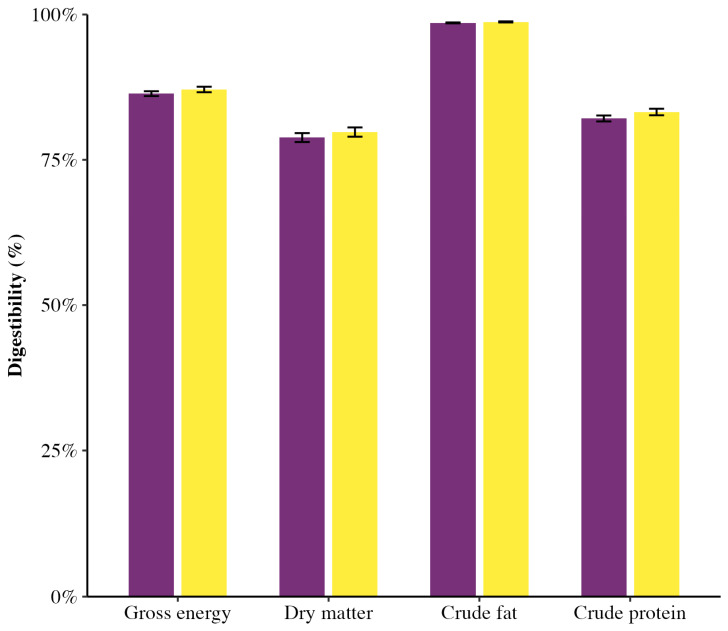
Apparent digestibility (%) for gross energy, dry matter, crude fat and crude protein in dogs wearing a coat (yellow bars) and dogs without a coat (purple bars). Error bars indicate standard error.

Apparent digestibility coefficients for gross energy and macronutrients under the two treatments (with coat and without coat) are shown in Figure 5. Apparent energy digestibility was similar between the no-coat (86.4 ± 0.4%) and coat-on periods (87.1 ± 0.5%; *p* = 0.117). Apparent dry matter digestibility also showed no differences between treatments (no-coat; 78.8 ± 0.8% vs. coat-on; 79.8 ± 0.8; *p* = 0.266). Crude fat digestibility also showed no differences (no-coat; 98.6 ± 0.1% vs. coat-on; 98.7 ± 0.1%; *p* = 114). Apparent crude protein digestibility, however, tended to be higher in the coat-on (83.2 ± 0.6% condition compared with the no-coat period (82.1 ± 0.5%; *p* = 0.079).

## 4. Discussion

This study aimed to evaluate the effects of wearing a coat overnight on the behaviour, activity and nutrient digestibility of New Zealand working farm dogs. While the wearing of coats did not significantly impact the apparent nutrient digestibility of the dogs, it did affect their sleep duration.

The average daily sleep time of the working dogs in this study was 11 h and 6 min. This is more than the previously reported 7.5 h for indoor kennelled research dogs [28], but less than the 13 h reported for middle-aged indoor kennelled research dogs [29]. It is, however, similar to shelter dogs who were reported to sleep for 11 h [30]. Differences in sleep duration between studies may have been influenced by housing conditions and associated daily routines. Direct comparisons between studies, however, are limited by methodological differences. Schork et al. [28] and Owczarczak-Garstecka and Burman [30] used similar video-based methodologies and definitions of sleep and rest, which differed from those used to train the machine learning model to classify dog behaviours in the current study [18], while Zanghi et al. [29] used an actigraphy-based sleep/wake classification approach.

Overall, dogs spent more time sleeping during the coat window (15:00–09:00 h) compared to the no-coat window (09:00–15:00 h). This finding reflects the expected diurnal activity patterns, with no differences observed between treatment groups outside the coat-associated window. In addition to environmental drivers such as light and temperature, predictable daily management routines, including the timing of staff presence, feeding, and husbandry activities, may also have contributed to the observed behavioural patterns. Animals housed under human-managed conditions frequently develop anticipatory behaviours in response to temporally predictable events [31], which can shape activity and rest cycles, and may partially explain the alignment of increased activity with staff arrival and reduced activity following their departure.

Wearing a coat increased sleep duration during the coat window, with a 10% increase in duration when the dogs were wearing the coat compared to that recorded when they were not wearing a coat. This difference in sleep duration was most evident between 18:00 h and 23:00 h, when the ambient temperature was cooling following sunset. The increased sleep duration when dogs wore a coat was likely due to the additional insulation. This insulation may have created a thermoneutral microenvironment that reduced the energy required to maintain core body temperature. Exposure to low ambient temperatures has been reported to elevate stress hormone levels and disrupt rest [32]. Therefore, coats may allow dogs to redirect metabolic resources toward restorative processes, with an increase in sleep as a primary outcome. In contrast, in the absence of coats, dogs may have engaged in subtle, energy-intensive thermoregulatory behaviours, such as shivering or higher metabolic heat production to maintain warmth [32], which could interfere with sustained sleep. Importantly, no adverse behavioural responses to coat use were observed in this study, which aligns with previous survey findings that indicated that working farm dogs generally showed high acceptance of coats when they were used [33]. This suggests that coat use is unlikely to negatively impact behaviour or welfare.

Variation in response to the coat treatment may also be influenced by inherent differences between dogs. Previous research in New Zealand has shown that Heading dogs can expend up to 23% more energy per kg metabolic body weight than Huntaways even when there are no differences in physical activity as measured with an accelerometer [3], suggesting underlying physiological differences between dog types. Additionally, variation in work type may influence optimal energy utilisation strategies (e.g., carbohydrate vs. fat metabolism) [34,35]. However, it should be noted that the dogs in the present study were not performing typical on-farm working tasks and followed a consistent daily routine with a set activity level. Therefore, the impact of breed-related differences in energy expenditure and metabolism on the study outcomes is likely to have been limited, although some individual variability cannot be excluded.

The average temperature during this study was 12 °C, which was below the thermoneutral zone for dogs (20–30 °C; [8]) but may still represent only a mild thermoregulatory challenge for healthy adult dogs under these conditions. Thus, the effect observed in this study may underestimate the true magnitude, and more pronounced responses may occur under colder environmental conditions. Additionally, thermoregulatory responses may vary between dogs depending on factors such as coat length and structure (e.g., single- vs. double-coated), with dogs possessing shorter or less dense coats potentially benefiting from external insulation at higher temperatures than those with more insulative coats [11,12]. New Zealand working farm dogs, such as Heading dogs, Huntaways and Harrier hounds, typically have short- to medium-length coats, which may provide less insulation compared to longer- or double-coated breeds. The typical New Zealand working farm dog may therefore be more susceptible to heat loss under cooler conditions, suggesting that they may particularly benefit from coat use in colder environments. However, more research is needed to confirm this.

Although sleep quality was not evaluated in the present study, previous research has shown that improved sleep can improve health, productivity, welfare, and emotional stability in working dogs [36]. Conversely, recent evidence suggests that not getting enough sleep may be linked to poorer welfare in dogs, with shorter periods of sleep associated with increased fear, anxiety, and stress-related behaviours [37]. Similar negative effects following sleep restriction, including disrupted normal behaviour and physiology, have also been observed in other animal species [38,39]. Evidence from human studies further supports the association between sleep and daily function. Among American adolescents, each hour of sleep disturbance was associated with a 3% decrease in the chance of engaging in daytime physical activity, and each hour of less sleep increased the chance of obesity by 80% [40]. Together, these findings suggest that getting enough sleep is important for both physical performance and overall wellbeing. However, the mechanisms underlying the observed increase in sleep duration remain speculative in the absence of physiological measurements. Variables such as core body temperature, cortisol, melatonin, or heart rate variability were not measured in the present study but could provide valuable insight into thermoregulatory and stress-related responses associated with coat use. For New Zealand farm dogs, which have high physical demands, thermoregulatory support may therefore help maintain both welfare and functional capacity.

Rest has been reported to be associated with metabolic processes in humans, including effects on hormone balance and energy metabolism [41]. However, there is currently limited evidence to support a direct effect of sleep on nutrient digestibility, particularly in dogs. In the current study, no differences in nutrient digestibility or metabolizable energy were observed between the no-coat and coat treatments. These findings were likely influenced by the relatively mild environmental conditions, which may have been insufficient to provide thermoregulatory or metabolic challenge. Under colder conditions, the insulating effect of a coat may become more apparent, as energy that would otherwise be used to maintain body temperature could be conserved and potentially allocated to other physiological processes, including digestion and nutrient utilisation [42]. In the present study, there was a small numerical increase in apparent protein digestibility when dogs wore coats; however, this effect was not statistically significant and should be interpreted with caution. This observation may reflect subtle changes in energy partitioning, where reduced thermoregulatory demand may decrease maintenance energy expenditure and allow greater allocation of energy toward digestive processes. However, given the small effect and lack of statistical significance, it is also possible that the difference in protein digestion reflects normal biological variation or a methodological artefact. Given the limited evidence linking sleep to nutrient digestibility, the underlying cause of the small numerical difference in protein digestion observed in this study remains unclear. Future studies conducted under colder environmental conditions, and with larger sample sizes, may help to clarify whether reducing thermoregulatory demand can influence nutrient utilisation in working dogs.

The current study employed triaxial accelerometers, which are a minimally intrusive technique that can capture animals’ natural activity–rest patterns while minimising experimental stress and promoting welfare [43]. Using a previously validated machine learning model with random forest analysis, we achieved a balanced accuracy of 0.94 in classifying sleep behaviour [18], providing a reliable reflection of the behavioural characteristics of the dogs. Despite these advantages, accelerometry remains limited because it records movement rather than brain activity and therefore cannot distinguish true sleep from quiet rest and it is unable to identify REM and non-REM sleep stages [44]. Therefore, sleep quality could not be assessed using this method. Given that dogs typically wake after REM sleep [45], advances in machine learning models may make it possible to accurately analyse canine sleep–wake cycles using this technology. Additionally, behaviours were classified into predefined categories. While other behaviours may have occurred, these would have been incorporated within broader classifications when they could not be reliably distinguished using accelerometry.

Conducting the experiment in the dogs’ natural living environment enhanced ecological validity but introduced potential confounders. Variations in wind speed, precipitation, and human activity could have influenced behaviour and perceived temperature, complicating the isolation of coat’s specific effects. Future research in a semi-controlled field setting could manipulate temperature, wind, and humidity to measure their independent and combined effects on sleep and thermoregulation. Controlled designs could also integrate core-body-temperature or heart-rate monitoring for a more direct assessment of thermal stress.

## 5. Conclusions

This study demonstrated that while the use of coats did not significantly affect nutrient digestibility, coat-wearing had a clear effect on sleep duration, with dogs spending more time sleeping when wearing coats, particularly during cooler periods. This suggests that providing insulation during the night may improve thermal comfort and support rest in outdoor-kennelled dogs. Although the study was conducted using research dogs under relatively mild environmental conditions, the findings provide promising evidence that coats may offer welfare and functional benefits for New Zealand working farm dogs. In practical farm settings, where dogs are exposed to colder conditions and higher physical demands, the use of coats may help reduce thermoregulatory strain, support recovery, and contribute to maintaining performance and body condition. Further research under real farm conditions, particularly during winter, is needed to confirm these effects.

## Figures and Tables

**Table 1:** Characteristics of dogs enrolled in the study, including breed, sex, age, body weight at the start of the study, and allocated treatment sequence.

Name	Breed	Sex	Age (years)	Body Weight (kg)	Phase 1 (7–11 Oct.)	Phase 2 (12–17 Oct.)
Buzz	Huntaway/ Bearded Collie	Male	3	24.9	Coat	No coat
Viva	Harrier Hound	Female	6	20.6	Coat	No coat
Cecco	Huntaway	Female	11	26.3	Coat	No coat
Ice	Heading Dog	Male	5	25.0	Coat	No coat
Bonnie	Huntaway	Female	5	24.5	No coat	Coat
Utah	Harrier Hound	Male	6	30.3	No coat	Coat
Archy	Harrier Hound	Male	5	26.0	No coat	Coat
Chloe	Harrier Hound	Female	5	22.5	No coat	Coat

**Table 2:** Macronutrient profile of the diet fed during the study.

	Amount per kg (as Fed)
Gross energy (kcal)	4756
Metabolizable energy (kcal) ^1^	4125
Dry matter (g)	921
Crude ash (g)	99
Organic matter (g) ^2^	822
Crude protein (g)	345
Crude fat (g)	195
Crude fibre (g)	21
Nitrogen free extract (g) ^3^	340

^1^ Metabolizable energy (kcal) =  [(GE_food_ − GE_faeces_) − ((g protein_consumed_ − g protein_faeces_) × 1.25)]/amount food consumed × 1000 [17]. ^2^ Organic matter = dry matter − crude ash. ^3^ Nitrogen-free extract = 1000 − crude ash − crude protein − crude fat − crude fibre.

## Data Availability

The raw data supporting the conclusions of this article can be made available by the authors on request.

## Abbreviations

The following abbreviations are used in this manuscript:

DBA	Dynamic body acceleration.
GLMM	Generalised linear mixed model.
ODBA	Overall dynamic body acceleration.

## Appendix A

Observed and model-predicted means ± standard error [95% CI] of time spent on sleeping, resting and active behaviours, and daily ODBA when dogs wore a coat and when they did not (Table A1).

**Table A1:** Observed and predicted means ± standard error [95% CI] of sleep, rest and active behaviours, and ODBA when dogs wore a coat and when they did not.

	Treatment	Observed	Predicted
Sleep (%)	No coat	40.2 ± 4.5 [29.6, 50.7]	40.0 ± 3.2 [33.8, 46.4]
	Coat on	48.1 ± 2.2 [43.0, 53.3]	48.1 ± 3.4 [41.6, 54.7]
Rest (%)	No coat	26.6 ± 3.1 [19.3, 33.8]	25.9 ± 2.7 [20.9, 31.6]
	Coat on	23.1 ± 2.4 [17.5, 28.6]	22.5 ± 2.5 [17.9, 27.8]
Active (%)	No coat	33.4 ± 3.9 [24.0, 42.3]	32.7 ± 3.1 [26.9, 39.1]
	Coat on	28.7 ± 2.6 [22.6, 34.8]	28.2 ± 2.9 [22.9, 34.3]
ODBA (Δg/24 h)	No coat	5798 ± 772 [3972, 7623]	5531 ± 760 [4225, 7240]
	Coat on	6034 ± 1181 [3241, 8827]	5464 ± 751 [4174, 7154]

## Appendix B

Observed and model-predicted means ± standard error [95% CI] of time spent on sleeping (%) for dogs wearing a coat versus not wearing a coat, across coat-associated time windows (coat window and no-coat window; Table A2).

**Table A2:** Observed and model-predicted means ± standard error [95% CI] of time spent sleeping (%) for dogs wearing a coat versus not wearing a coat, across coat-associated time windows (coat window and no-coat window).

Coat Condition	Coat-Associated Time Window	Observed	Predicted
No coat	No-coat window	9.7 ± 2.1 [4.8, 14.6]	9.8 ± 2.0 [6.5, 14.4]
	Coat window	49.4 ± 5.3 [36.9, 61.9]	49.4 ± 4.2 [41.2, 57.6]
Coat on	No-coat window	9.8 ± 2.8 [3.1, 16.5]	8.8 ± 1.9 [5.7, 13.2]
	Coat window	60.0 ± 2.5 [54.1, 66.0]	60.1 ± 4.1 [51.8, 67.8]

## Appendix C

Observed and model-predicted means ± standard error [95% CI] of time spent on resting (%) for dogs across coat-associated time windows (coat window and no-coat window; Table A3).

**Table A3:** Observed and model-predicted means ± standard error [95% CI] of time spent resting (%) for dogs across coat-associated time windows (coat window and no-coat window).

Coat-Associated Time Window	Observed	Predicted
No-coat window	47.3 ± 6.3 [32.3, 62.3]	47.0 ± 5.1 [37.4, 56.9]
Coat window	18.0 ± 1.6 [14.1, 21.8]	17.2 ± 3.2 [11.7, 24.5]

## Appendix D

Observed and model-predicted means ± standard error [95% CI] of time spent on active behaviours (%) for dogs across coat-associated time windows (coat window and no-coat window; Table A4).

**Table A4:** Observed and model-predicted means ± standard error [95% CI] of time spent on active behaviours (%) for dogs across coat-associated windows time windows (coat window and no-coat window).

Coat-Associated Time Window	Observed	Predicted
No-coat window	42.9 ± 5.4 [30.1, 55.8]	43.1 ± 4.5 [34.7, 52.0]
Coat window	27.4 ± 3.8 [18.5, 36.4]	27.8 ± 4.0 [20.7, 36.3]

## Appendix E

Observed and model-predicted means ± standard error [95% CI] of daily ODBA (Δg) for dogs wearing a coat versus not wearing a coat across coat-associated time windows (coat window and no-coat window; Table A5).

**Table A5:** Observed and model-predicted means ± standard error [95% CI] of daily ODBA (Δg) for dogs wearing a coat versus not wearing a coat across coat-associated time windows (coat window and no-coat window).

Coat Condition	Coat-Associated Time Window	Observed	Predicted
No coat	No-coat window	559 ± 110 [298, 819]	503 ± 71 [383, 663]
	Coat window	178 ± 25 [120, 236]	170 ± 24 [129, 223]
Coat on	No-coat window	600 ± 114 [330, 870]	546 ± 76 [415, 718]
	Coat window	161 ± 22 [108, 214]	153 ± 21 [116, 201]
